# Intensive 2-days training on perfused human placenta for microvascular anastomoses

**DOI:** 10.1007/s00701-024-06286-6

**Published:** 2024-11-15

**Authors:** Elisa Colombo, Fabian Wolf, Fiona Helg, Lara Höbner, Jennifer A. Watson, Martina Sebök, Christian Haslinger, Tristan van Doormaal, Luca Regli, Giuseppe Esposito

**Affiliations:** 1 Department of Neurosurgery and Clinical Science Center, University Hospital Zurich, Zurich, Switzerland; 2 Department of Plastic and Hand-Surgery, University Hospital Zurich, Zurich, Switzerland; 3 Department of Obstetrics, University Hospital Zurich, Zurich, Switzerland; 4 Department of Neurosurgery, University Medical Center Utrecht, Utrecht, the Netherlands

**Keywords:** Microsurgery, Microanastomosis, Placenta, Training

## Abstract

**Background and Purpose:**

We report on an intensive two-day training program on microanastomoses performed on perfused human placenta models. A specific scoring system was elaborated to evaluate the participants’ microsurgical skills and report the participants’ results.

**Materials and Methods:**

Trainees who attended the Zurich Microsurgery Courses in 2023 were included in the study. Before performing the microanastomoses, each participant received a visual didactic training. Training was made on perfused human placenta models. To perform the microvascular anastomoses, vessels of different diameters were chosen, and 9–0 and 10–0 microsutures were used. The course was structured in two days. On day one, participants practiced microvascular dissection, microsuturing and end-to-end anastomoses, while the second day was dedicated to end-to-side and to repeat the most useful microanastomosis depending on the specialty. A score system for the evaluation of a successful microanastomosis was developed and applied to assess the participants’ anastomoses. User satisfaction was measured by means of a survey-based questionnaire.

**Results:**

Fifty-two participants from different institutions, specializations and levels of experience were included. A significant improvement in the overall microsurgical skills of the included cohort was documented (*p* < 0.005). The initial average score per anastomosis of 3.56 points (SD 0.71) increased to an average of 3.8 points (SD 0.87) at the end of the course. The steepest learning curve was observed in the placement of knots (Δ 0.48 points, *p* = 0.003) and microvascular dissection (Δ 0.44 points, *p* = 0.002). Most participants rated the fidelity and importance of the placental microsuturing course as extremely high.

**Conclusion:**

The two-day training program is efficient to teach microvascular dissection and microanastomosis techniques. A significant improvement of participants’ microsurgical skills was reported. The human placenta model proved to be a high-fidelity simulator with great user satisfaction.

## Introduction

The successful performance of a microsurgical vascular anastomosis (MVA) is a technically demanding task which requires extensive practice [13, 14]. The ideal training strategy in terms of length of training and tools used for training is yet to be found [14]. As timing of training is concerned, few studies have endorsed training sessions distributed in time [14, 16]. Plastic models and animal models are commonly used for the training of MVAs. Plastic models (silicone microtubing) and non-live animal models such as chicken legs show a limited similarity to the in vivo conditions [1]. Furthermore, the use of live animal models becomes increasingly difficult and its ethicality is often questioned [5, 7].

The placenta model holds potential for the training of MVAs as it has been found to be superior to plastic materials and animal models for microsurgical training concerning availability and costs [5]. Further, the perfused placenta model provides a realistic setting compared to the intraoperative. conditions [1, 7]. Arteriotomies, microvascular dissection (MVD), microsuturing and end-to-end, end-to-side, and side-to-side microvascular anastomoses can be practiced on this model using multiple vessels of different diameters.

The objective of the present study was the endorsement of the use of the perfused human placenta as efficient and high fidelity model to practice MVD and MVA techniques in an intensive two-day training strategy, and the assessment of the learning curve during the training as well as the user satisfaction with the model.

## Materials & methods

The placenta is obtained from the Obstetrics Department of the University Hospital Zurich, Switzerland and the mother signed an informed written consent form beforehand. During pregnancy, tests for common infectious diseases are carried out, and potentially infectious specimens are not selected for training, as well as specimens requiring pathological study. Only placentas that would have been discarded as medical waste have been used.

### Subjects

Participants with varying microsurgical background and experience attending the Zurich Microsurgery Course were included in this study. The participants were resident in training or specialists in different surgical fields that rely on microsurgical techniques, mainly neuro-, hand-, plastic-, ear nose and throat (ENT)-, oral-maxillofacial surgery. Therefore, the group presented heterogeneous in respect to age, level of training and country of origin. All participants voluntarily agreed to take part to the study. The participants were retrospectively categorized according to their experience and assigned to two groups depending on the total amount of surgical traineeship at the time of the course.

- Group 1: 0–5 years of experience
- Group 2: > 5 years of experience

Experience was defined as years of activity in specialty (including years spent for residency). Statistical analyses were conducted to investigate any improvement of the microsurgical skill level through a comparative analysis and to document the learning curve between the groups.

### Training material

To perform the microvascular anastomoses, human placentas were used. Perfused placenta specimens were prepared as reported in details elsewhere from our group.^7^ Briefly, placentas were rinsed and the parietal amniotic sac was removed. The umbilical cord was cut, and the central vein was cannulated in the cutting plane. The two arteries were punctured more distally at the base of the umbilical cord and all vessels were flushed with non-diluted heparinised saline. The finished models were frozen at -80° Celsius until use for the course. Placental vessels of different diameters ranging from 1 to 4 mm were chosen, and 9–0 and 10–0 sutures were used.

### Training protocol

Subjects participated in a two-day microsurgery hands-on on perfused placenta models. Before practical training of the microsurgical skills, participants received a visual didactic training session concerning equipment, general microsurgical and MVD and MVA types and techniques. The half of day 1 was dedicated to practice microvascular dissection and microsuturing. The second part of day 1 was focused on end-to-end anastomoses, while the second day was dedicated to end-to-side and to repeat the most useful microanastomosis depending on the specialty.. At the end of both days, all completed anastomoses were rated by a board-certified neurosurgeon with more that 10-year experience in bypass surgery (G.E.) according to the suggested scoring system.

### Score system

The proposed score system considered 8 parameters, which exemplify the elements of a successful microvascular anastomosis. Table 1 provides a list of the parameters comprised in the scoring system and their definition as established by the senior authors (GE, TvD, LR, JAW).

**Table 1:** Schematization of the parameters composing the score system used during the course and their definitions

Parameters of patent microsurgical anastomosis:	
Borders:	Adequacy of border adaption
Number of Knots:	Correct number of knots (end-to-end: 8–12 sutures; end-to-side: 10–14 sutures)*
Symmetry of knots:	Knots were put in symmetrical distances, similar number on all sides
Precision of sutures:	Symmetrical distance from vessel border, all vessel layers were used
Threads:	Adequate length of cut threads – threads outside the lumen
Patency:	Visual documentation of complete lack of obstruction after perfusion of the anastomosis with saline solution
Leak:	Leaks due to holes after perfusion of the vessel
Microvascular dissection:	Adequate dissection/preparation of microvessels and the tissue in the vicinity of anastomosis

*‘Number of Knots’ was evaluated in accordance to the diameter of the vessel(s) used for the microanastomosis, which was noted for every exercise

For each item, the trainee received a score between one (minimum) and a maximum of five points. As shown in Table 2, one point was given if 0–20% of the task was correctly achieved, and two points if between 21 and 40%.Three points were awarded if 41–60% of the task was correctly performed, and four points if correctness of performance lied between 61 and 80%. A maximum of five points was given for flawless execution of the task with a correctness close to 100%.

**Table 2:** Visual summary of the strategy used to assign a numerical evaluation to each parameter of the score

Points given	Percentage (%) of correctness
1/5 (minimum)	0–20%
2/5	21–40%
3/5	41–60%
4/5	61–80%
5/5 (maximum)	> 81% up to 100%

The parameter `Border` was graded according to the percentage of correctness of vessels’ edges approximation.

The parameter `Number of Knots’ was evaluated in accordance to the diameter of the vessel(s) used for the microanastomosis, which was noted for every exercise.

For the parameters ‘Symmetry of knots’, ‘Precision of sutures’, and ‘Threads’, the score was evaluated based on the percentage of correctness of the specific task. For instance, a microanastomosis with 10 sutures was given a score of 4 out of 5 on ‘Threads’ if 7 sutures had an adequate length of the cut threads.

The parameter `Microvascular dissection` was evaluated according to the percentage of the anastomosed vessels’ surface which was properly dissected.

#### User satisfaction

At the end of each course the participants documented their satisfaction after using the model and rated the importance of placental microsurgical training by means of a questionnaire. The feedback form consisted of four questions with answers ranging from five (very important/very similar) to one point (not important/not similar). Table 3 presents the questionnaire on satisfaction:

**Table 3:** Questionnaire on user’s satisfaction provided to each trainee at the end of the course

Questions:	Possible ratings:				
«Is the live vessel dissection similar to the placenta simulator?»	Very similar	Similar	Moderately similar	Slightly similar	Not similar
«What is the degree of importance of the placenta simulator?»	Very important	Important	Reasonably similar	Slightly important	Not important
«Do you think placenta continuous* training is important if you had microsurgery practice?»	Very important	Important	Reasonably important	Slightly important	Not important
«Do you think placenta continuous training is important if you did not have any prior microsurgical training practice?»	Very important	Important	Reasonably important	Slightly important	Not important

*continuous: in these settings it means repeated training with the model

### Statistical analysis

All statistical analyses were performed using R Studio (RStudio, PBC, Boston). Data were presented as numbers and percentages, means and standard deviations (SD). Performance improvement inside the groups was calculated by means of a paired Student’s t-test. To compare inter-group performance a two-sided Welch’s t-test was implemented.

## Results

### Subjects

A total of 52 practicing residents, junior and senior surgeons participated in this study. Overall, the cohort presented an average surgical experience of 6.4 years (SD 4.7 years, range 0–20 years). Most participants were neurosurgeons (*n* = 36, 69%), four participants had a background in ENT (8%), each three participants in hand (*n* = 3, 6%) and plastic surgery (*n* = 3, 6%). Nine subjects (17%) had prior experience in microsurgical training courses while only four surgeons (8%) had performed microvascular anastomoses in patients before. 26 participants (50%) had ≤ 5 years of experience and were retrospectively assigned to subgroup one. The same number of participants (26/52, 50%) had a surgical experience ≥ 6 years, and they were assigned to subgroup two. Table 4 summarizes the participants’ information and characteristics:

**Table 4:** Visual summary of the participants’ information and characteristics

	*n* in % (*n* = 52)
Male gender	58%
Specialization of participants:	
Neurosurgery	69%
ENT	8%
Hand surgery	6%
Plastic surgery	6%
General surgery	4%
Orthopaedic surgery	4%
Maxillofacial surgery	4%
Experience in surgical traineeship:	
≤ 5 years	50%
Experience with microscopes and MVD and MVAs:	
Regularly used microscope for operation	71%
Prior experience in microsurgical training courses	17%
MVAs performed on patients	8%

### Cohort improvement

**Fig. 1:**
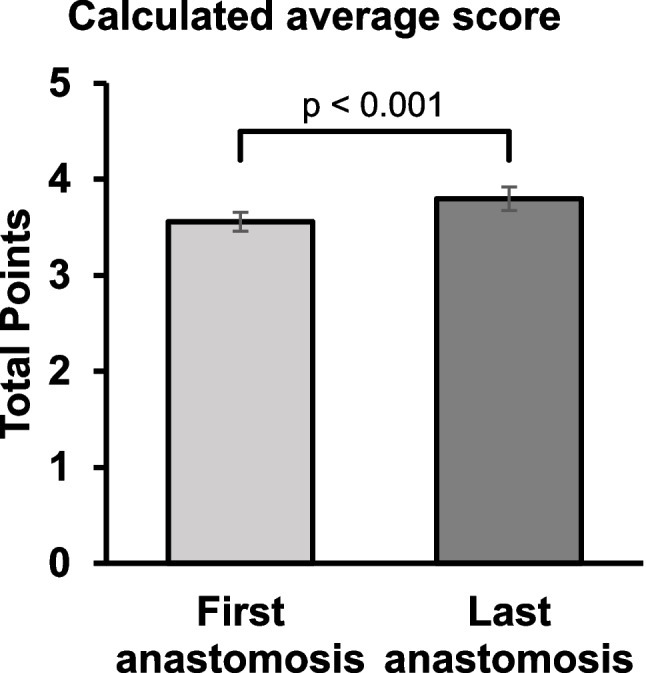
Visualization of the comparative analysis between the average score obtained by the participants’ cohort on their first anastomosis and on the last one

The participants performed an average 3.9 anastomoses (SD 1.33) during the two-day course. The score for their first anastomoses averaged at 3.56 points (SD 0.71). The calculated score for the last anastomosis came to an average of 3.80 (SD 0.87), with a difference of 0.34 points (*p* < 0.001). Figure 1 provides a visualization of the comparative analysis between the average score obtained by the participants’ cohort on their first anastomosis and on the last one.

The steepest learning curve was observed for the placement of knots and handling of the microsuturing threads. The initial average group score for «precision of knots» was 3.50 points (SD 0.83) and improved to a final average score of 3.98 points (SD 0.95), equalling to an overall increase of 0.48 points (*p* = 0.003). In the category «threads» the participants scored an average of 3.21 points (SD 0.87) for the first sutured anastomosis. With the last anastomosis performed the group achieved a mean of 3.63 total points (SD 1.20). The overall increase with 0.42 points was statistically significant (*p* = 0.044).

The skill with the least amount of improvement between first and last anastomosis was the total «number of knots» needed. Participants started with an average score of 3.83 points (SD 1.02), the mean after completing the course amounted to 3.90 points (SD 1.12). Therefore, a difference of 0.08 points (*p* = 0.81) between the first and last anastomosis was documented.

Table 5 summarizes the average scores achieved at beginning and by the end of the course:

**Table 5:** Summary of the the average scores achieved at beginning and by the end of the course by the whole cohort of participants

Subscores	∅ score (points) first anastomosis	∅ score (points) last anastomosis	Mean difference (points) between start and end	*p*-value
Borders	3.38 (SD 0.91)	3.57 (SD 1.25)	0.19	0.42
Number of Knots	3.83 (SD 0.99)	3.90 (SD 1.14)	0.07	0.81
Symmetry	3.50 (SD 0.83)	3.69 (SD 1.16)	0.19	0.36
Precision of Knots	3.50 (SD 0.83)	3.98 (SD 0.95)	0.48	0.003
Threads	3.21 (SD 0.87)	3.63 (SD 1.20)	0.42	0.044
Patency	4.06 (SD 1.08)	4.18 (SD 0.99)	0.12	0.74
Leak	4.02 (SD 1.19)	4.0 (SD 1.08)	-0.02	0.68
Adventitia preparation	2.98 (SD 0.87)	3.42 (SD 0.84)	0.44	0.002

### Improvement according to years of experience

When comparing the total number of calculated points between the first and last sutured anastomosis no significant difference between the experienced and inexperienced participants was visible. For the initial anastomosis, group 1 (less experienced) scored an average of 3.61 points (SD 0.6) while group 2 (more experienced) reached a total average of 3.51 points (SD 0.81). A p-value of 0.06 was detected when comparing the first anastomoses of the two subgroups with each other.

Analysing the individual tasks composing the overall score, group 1 tended to perform marginally better in the first anastomosis, while group 2 averaged a higher score in the last anastomosis. Nonetheless, a statistical significance was not observed. On the last anastomosis group 1 scored 3.74 points and group 2 3.86 points. The difference of the score of the last anastomoses of the two subgroup was at a p-value of 0.7.

**Fig. 2:**
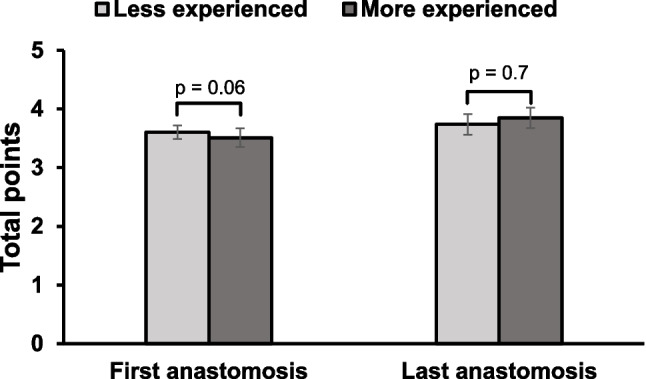
Visualization of the average scores achieved for first and last anastomosis according to the subgroups

Figure 2 visualizes the average scores made for first and last anastomosis according to the subgroups.

Examining the learning curve inside the two subgroups, differences could be appreciated. While for group 1 neither the total calculated score nor any of the subscores showed a significant increase, group 2 showed significant skills improvement. The average total calculated score increased from initially 3.51 points to 3.85 points in the last anastomosis, a difference of 0.34 points (*p* < 0.001). The most notable improvement of subgroup 2 was seen in the categories «adventitia preparation» (Δ 0.67, *p* = 0.003), «threads» (Δ 0.73, *p* = 0.001) and «knots» (Δ 0.58, *p* = 0.008).

Table 6 summarizes the scores achieved in the different items.

**Table 6:** Summary of the average scores achieved at beginning and by the end of the course by Group 1 (less experiences participants) and Group 2 (more experienced participants)

Subscores according to group:				
Group 1	Ø score first anastomosis	Ø score last anastomosis	Mean difference (points) between start and end	*p*-value
**Borders**	3.46 (SD 0.86)	3.64 (SD 1.25)	0.18	0.77
**Number of Knots**	3.77 (SD 0.86)	3.80 (SD 1.22)	0.03	0.87
**Symmetry**	3.50 (SD 0.81)	3.60 (SD 1.22)	0.1	0.87
**Precision of Knots**	3.58 (SD 0.76)	3.96 (SD 0.98)	0.38	0.133
**Threads**	3.27 (SD 0.87)	3.36 (SD 1.25)	0.09	0.90
**Patency**	4.04 (SD 0.96)	4.08 (SD 1.08)	0.04	0.87
**Leak**	4.04 (SD 1.11)	4.08 (SD 0.91)	0.04	0.89
**Microvascular Dissection**	3.19 (SD 0.75)	3.40 (SD 0.76)	0.21	0.18
**Group 2**				
** Borders**	3.31 (SD 0.97)	3.50 (SD 1.27)	0.19	0.35
** Number of Knots**	3.88 (SD 1.18)	4.0 (SD 1.02)	0.12	0.59
** Symmetry**	3.50 (SD 1.14)	3.77 (SD 1.11)	0.27	0.26
** Precision of Knots**	3.42 (SD 0.90)	4.0 (SD 0.94)	0.68	0.008
** Threads**	3.15 (SD 0.88)	3.88 (SD 1.11)	0.73	0.001
** Patency**	4.08 (SD 1.22)	4.27 (SD 0.92)	0.19	0.56
** Leak**	4.00 (SD 1.29)	3.92 (SD 1.23)	- 0.08	0.66
** Microvascular Dissection**	2.77 (SD 0.95)	3.44 (SD 0.92)	0.67	0.003

### User satisfaction

A total of 49 subjects (49/52, 94%) completed the questionnaire at the end of the second day of the course.

The majority of participants perceived the placental dissection to be very similar (18/45, 40%, 5/5 points) or similar (18/45, 40% 4/5 points) to an actual vessel dissection.

44 subjects (44/49, 90%) rated the importance of the placental lab exercise with 5/5 points (very important). The remaining five participants (5/49, 10%) perceived the dissection as important (4/5 points).

A total of 27 participants (27/39, 69%) valued the placental dissection as very important (5/5 points) and nine people (9/39, 23%) assessed the importance of a continuous training as 4/5 points (important). Two participants (2/39, 5%) defined as ‘reasonably important’ the continuous training with the placenta simulator (3/5 points), whereas only one participant (1/39, 3%) defined it as of ‘poor importance’.

With 26 subjects (26/38, 68%) the majority rated an extended training as very important (5/5 points) and nine people (9/38, 24%) thought it to be important (4/5 points).

**Fig. 3:**
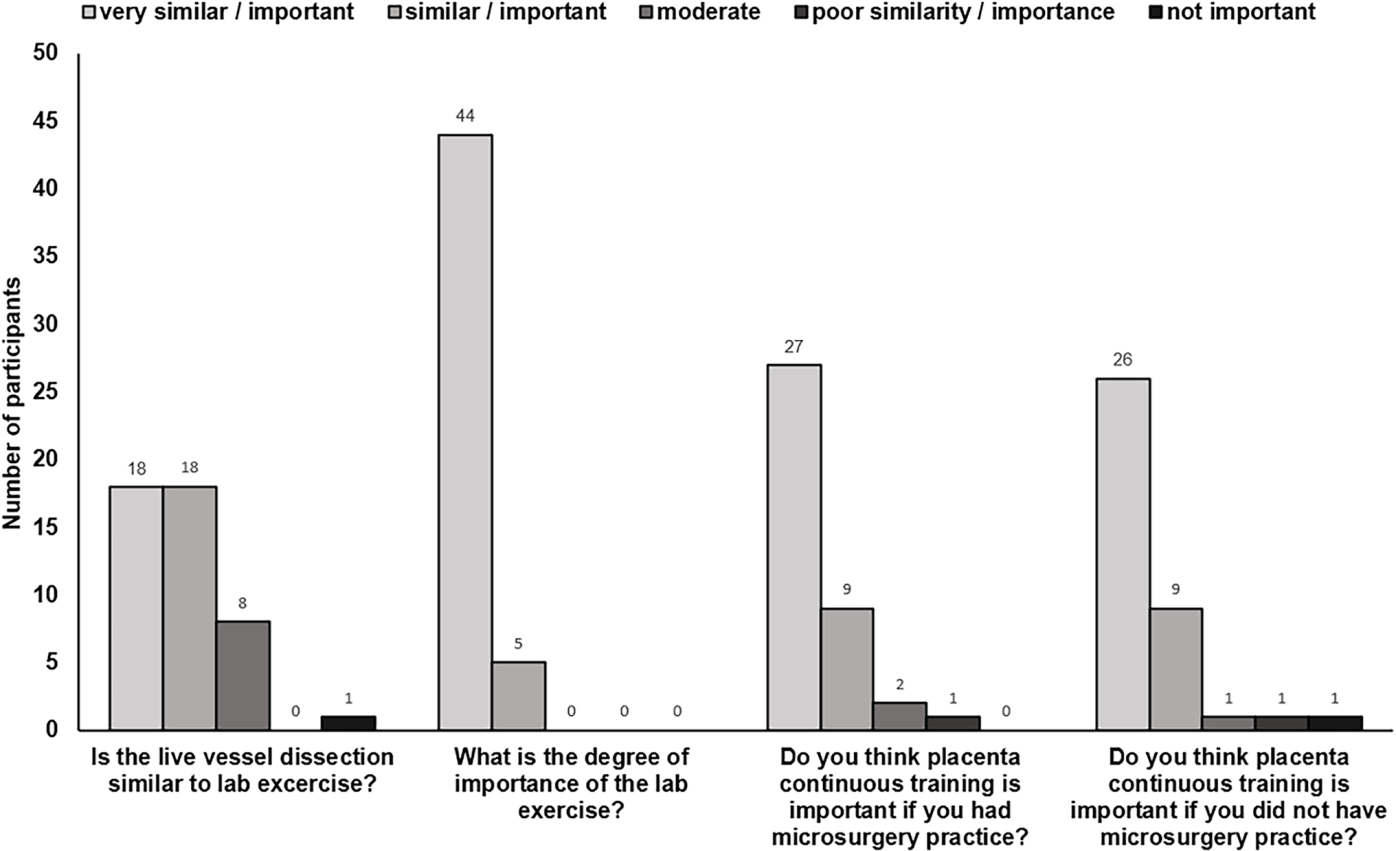
Visual representation of the results of the user satisfaction questionnaires

Figure 3 presents the results of the user satisfaction questionnaire.

## Discussion

The present study aimed to endorse the use of the perfused placenta as a model to practice MVAs during the intensive two-day Zurich Microsurgery Course. The learning curves of 52 practicing surgeons were analyzed using the specific scoring system developed in alignment with the course. The aim of the scoring system was to provide the trainees with an accurate and detailed assessment of their microsurgical skills.

Microvascular surgery is a high-skill subspecialty that requires extensive training as intraoperative complications can lead to devastating impairment of the patient. High-fidelity models are necessary to provide neurosurgical residents as well as residents from other specialties like plastic and hand surgery, maxillofacial surgery and young attendings with adequate exercise. While in vivo animal models could be still considered gold standard for learning microsurgical techniques – as they reproduce a physiological environment and are able to simulate intra- and postoperative complications [20] – they slowly lose popularity because of their ethical and economical controversies. Low-cost, high-availability models such as synthetic tubes or human/animal placenta have gained increased success over the last few years.

The use of human placenta as a viable model for vascular microsurgery laboratory training was previously established [2, 4, 5, 17, 19]. Different studies highlighted the possibilities to simulate various neurosurgical bypass techniques using placental vessels, content and construct validation was established regarding replication of intracranial-intracranial and extracranial-intracranial bypass operations [6, 18]. Further refinement of the model allows for simulation of pulsatile vascular behavior and intraoperative vessel rupture [3, 9]. Furthermore, the predictive validity of human placentas for aneurysm microsurgical training was established [4, 12].

In this manuscript we also report on the use of a novel score based on the most relevant parameters defining a successful microvascular anastomosis. The score is used to assess the correctness of a performed microanastomosis.

Using the score, a significant improvement of surgical skills was found after the completion of the intensive two-day Zürich Microanastomosis course for participants at all levels of experience. Regarding the various assessed subskills, the "leak" category was the sole area where a marginal decline in scores was recorded at the course's conclusion. Additionally, the subskill "number of knots" exhibited minimal progress, potentially due to the participants' initial high average score of 3.83 points. In contrast, subskills such as MVD, knot precision, and ‘number of suture’, which posed greater challenges, were particularly demanding for participants during initial anastomoses, thus leading to lower initial scores. With more practice throughout the course, trainees could concentrate on these specific tasks, leading to a significant enhancement of their skills.

Subgroup analysis showed no significant difference in scored points between experienced and inexperienced participants. Initial scores were closely matched, and both groups showed marginal variations in performance across different anastomoses, though none reached statistical significance. The learning curve analysis within each subgroup revealed that, while group 1 did not demonstrate statistically significant improvements, group 2 showed a notable enhancement in microsuturing skills, evidenced by a relevant increase in their average score from 3.51 to 3.85 in the final anastomosis. Particularly, group 2 exhibited substantial advancements in "microvascular dissection," "threads," and "number of knots" categories, highlighting the impact of experience on specific microsuturing competencies.

Most participants rated the practice of MVAs on the perfused human placenta as “important” or “very important”. The majority of participants found placenta continuous training was “important” or “very important”. Face validity of the perfused human placenta was found to be high overall with 80% answering it was “similar” to “very similar” to a live vessel dissection. These findings are consistent with literature, which has already demonstrated that the human placenta model has a high face, content and construct validity. Recently the human placenta model has emerged as an utmost important tool to learn and improve microsurgical skills [8, 19]. Multiple studies refined the techniques to match these simulations to in vivo situations [10, 11, 21]. Survey-based questionnaires concluded the same results as this study and assessed the human placenta model as a high-fidelity simulator that achieves maximal similarity to in-vivo tissue and surgical situations. User feedback concerning the implementation of placental models in standard microsurgical training courses matched the high demand that was observed in our course [5].

While previous studies documented the value of distributed training sessions for microvascular anastomoses [15, 16], the present study was able to show a significantly improved learning curve after an intensive two-day course. Therefore, a short intensive training scheme may provide the necessary time to transfer theoretical knowledge about different surgical techniques, tips and tricks. Subsequent distributed practice may complement an initial massed course by strengthening the acquired expertise and potentiate long-term muscle-memory and skill retention.

This study demonstrated a significant enhancement in microsurgical skills following the two-day Zurich Microsurgery Course. However, its findings are tempered by limitations such as a relatively small sample size and a partially retrospective approach in comparing the competencies of experienced and inexperienced participants, which failed to reveal a marked difference between these two groups. On average, each participant completed four microanastomoses distributed across end-to-end and end-to-side subtypes. Notably, the first and last anastomoses performed during the course typically varied in type due to the different techniques being practiced on separate days, introducing a potential confounding factor in the form of varying complexity among the examined anastomoses that could attenuate subtype-specific improvements.

Another challenge to the study's integrity is the subjective bias inherent in the anastomosis assessment process. Although the scoring system proposed is designed to be objective, the evaluation was conducted by only two surgeons (GE, JAW), who were not blinded to either the participants or the number of attempts made, possibly influencing the outcomes. Furthermore, the scoring system did not include the timing to perform the MVA, which should represent a future integration to the score given the relevant role of Timing in the real clinical practice. To address these concerns and further validate the scoring system and the associated learning curve, subsequent iterations of the Zurich Microsurgery Courses will include enhancements to the study's design and methodology.

## Conclusion

This study demonstrates an increased learning curve on performance of microsurgical anastomosis after an intensive two-day training program on human placental vessels. The placenta proves to be a high-fidelity model with satisfactory user experience, providing high similarity to in-vivo situations without the use of animal models. The scoring system developed is easily implementable and able to track significant improvement in different subskills of microvascular surgeons. Microsurgery Course using placenta as simulators offer an excellent environment to familiarize and improve neurovascular microsurgical skills to all levels of surgical experience.

## Data Availability

No datasets were generated or analysed during the current study.
